# 

**Published:** 1904-11-15

**Authors:** 


### ANNOUNCEMENTS.

THE Kentucky State Board of Examiners will meet in Louisville, December 6th, for the examination of persons desiring to practice in the State. Application must be made to the secretary fifteen days before the meeting. Candidates must be graduates of reputable dental colleges.

C. R. SHACKLETTE, *Secretary*,

628½ *Fourth Ave., Louisville, Ky.*

THE Hartford (Conn.) Dental Society held its annual meeting October 10, 1904, and elected Dr. G. M. Griswold, President; Dr. C. E. Barrett, Vice-President; Dr. A. E. Cary, Secretary; Dr. E. R. Whitford, Treasurer; Drs. Munger, Youngblood and Hunt, Executive Committee; Dr. Dryhurst, Librarian, and Dr. Henry McManus, Historian.

Dr. Guy Blencoe, of Alma Center, Wisconsin, was married October 25, 1904, to Miss Olive Miller. We are glad to extend our hearty congratulations and best wishes for happiness and prosperity.

Dr. James A. Dale is now editor of *The Dental Headlight.* His sterling character, natural endowment and education, with a genius for hard work and willingness to sacrifice, qualify him for a high place in this work.

The Arkansas State Board of Examiners will meet December 2d and 3d, in Little Rock. Candidates must come prepared to fill a tooth and pass a written and oral examination on all usual subjects. Examination fee is $5.00. No temporary licenses will be given to any one. Full particulars may be had by addressing Dr. A. T. McMillin, Little Rock, Arkansas.

The thirty-ninth annual meeting of the Ohio State Dental Society will be held in Columbus, Ohio, on the 6th, 7th and 8th of December, at the Great Southern Hotel. A good program is in preparation, and a good meeting will surely follow. The clinic committee is actively at work securing clinics that will interest as well as instruct all classes of practitioners. Any one having a suitable subject to present for a clinic should address Dr. C. A. Hawley, Y. M. C. A. Building, Columbus, for particulars as to arrangement of time and space.

